# Retraction: LINC01315 impairs microRNA-211-dependent DLG3 downregulation to inhibit the development of oral squamous cell carcinoma

**DOI:** 10.3389/fonc.2024.1378198

**Published:** 2024-07-04

**Authors:** Frontiers Editorial Office

The journal retracts the article cited above.

Following publication, concerns were raised regarding the integrity of the images in the published figures.

The authors failed to provide a satisfactory explanation during the investigation, which was conducted in accordance with Frontiers’ policies. As a result, the data and conclusions of the article have been deemed unreliable and the article is retracted.

This retraction was approved by the Chief Editors of Frontiers in Oncology and the Chief Executive Editor of Frontiers. The authors do not agree to this retraction.

